# Tocilizumab in the Treatment of Eosinophilic Fasciitis: A Case Study and Review of Literature

**DOI:** 10.31138/mjr.34.1.78

**Published:** 2023-03-31

**Authors:** Sarah El Iskandarani, Maya Khasho, Mira Merashli

**Affiliations:** 1 Memorial Sloan Kettering Cancer Center, Integrative Medicine Service, New York City, New York, USA,; 2 Tishreen University, Faculty of Medicine, Latakia, Syria,; 3 American University of Beirut, Department of Internal Medicine, Division of Rheumatology, Beirut, Lebanon

**Keywords:** eosinophilic fasciitis, Shulman’s disease, tocilizumab

## Abstract

Eosinophilic fasciitis (EF) is a rare connective-tissue disorder that is characterised by subacute onset of erythema, oedema, and induration of the skin and soft tissues of the limbs and trunk. Although several triggers have been hypothesised to be associated with EF, the aetiology of eosinophilic fasciitis (EF) is still unclear, and several treatment regimens have been proposed to treat this disease. In this article, we report a case of a 72-year-old gentleman with multiple comorbidities who presented to the clinic for diffuse skin thickening present on his forearms, thighs, legs bilaterally, and over the pelvis. The patient was diagnosed with EF and failed multiple treatment regimens including prednisone, methotrexate, rituximab, but finally responded and was maintained on tocilizumab. In this article, we will review the current understanding of EF, diagnostic approach, popular treatments and review other cases of EF in which tocilizumab was used.

## INTRODUCTION

Eosinophilic fasciitis (EF) is a rare connective-tissue disorder that was primarily described by Dr. Shulman in 1974 as a scleroderma-like syndrome.^1^ The disease is characterized by subacute onset of erythema, oedema, and induration of the skin as well as soft tissues of the limbs and trunk.^1,2^ EF classically presents with progressive thickening of the skin associated or in some cases can present with arthralgias.^3^ Additionally, EF could be associated with malignancies.^3^ Peripheral eosinophilia is quite common in these patients, but is not needed for diagnosis.^3^ Even though several triggers have been hypothesized to be associated with Shulman’s disease, the aetiology of eosinophilic fasciitis (EF) is still unclear.^4^

## CASE DESCRIPTION

### Chief Complaint and History Taking

A 72-year-old retired gentleman, heavy smoker, known to have hypertension, diabetes mellitus type II, and chronic obstructive pulmonary disease presented to the clinic for diffuse skin thickening. This thickening was present on his forearms, thighs, and legs bilaterally, as well as his pelvis. This symptom started 8 months prior to presentation and was associated with excessive itching. The patient reported 8 kg weight loss in the last 45 days which he attributed to decreased appetite. The patient denied any joint pain or swelling, Raynaud’s or sicca symptoms, morning stiffness, fever, or night sweats. He previously presented to other healthcare centres and tried deflazacort 30 mg once daily for 20 days and ebastine 20 mg, but with no improvement. The patient denies family history of rheumatological diseases. As for social history, the patient smoked 10 cigarettes per day for 20 years. He has no pets, no recent travel, and no history of drug abuse. He did not report engaging in harsh exercise or jobs in the past.

### Physical Exam

Physical exam was pertinent for skin hardening over the flexor surface of both forearms, back, lower abdomen, thighs, and legs. Hypopigmentation was present over the left side of the face and left upper axillary line. Multiple non-tender cervical lymph nodes could be palpated with a fibrosed texture. No muscle or joint tenderness was present.

### Lab studies

Initially, patient had a WBC 10.60×10^3^/mm^3^ with the percentage of eosinophils being 13.2% (1.4×10^3^ absolute eosinophilic count). The rest of the complete blood count with differential was normal. CRP was 10 mg/dl (N=0–1). ESR was 10 and 22 after 1 hour and two hours respectively (N=1–20). C3, C4 were within normal levels.

**Figure 1.**
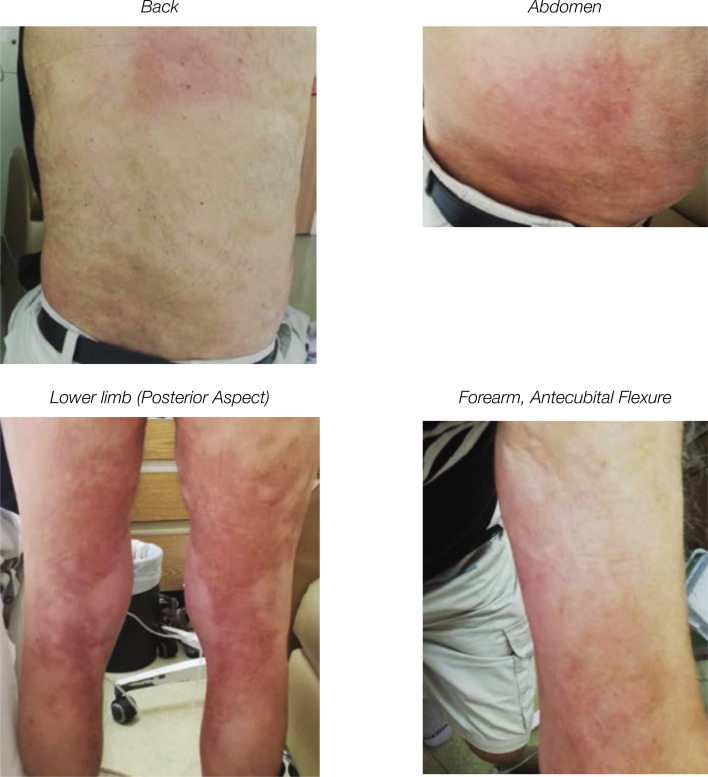
Physical exam findings before treatment.

ANA, RA latex, anti-dsDNA, anti-SSA, anti-SSB were all negative. Renal, liver, and thyroid function tests were normal. CPK was normal on different occasions. SPEP showed no monoclonal gammopathy.

### Imaging

CT of the chest, abdomen, and pelvis was done to rule out an underlying malignancy.

### Skin biopsy result

Muscle and facial biopsy done in another institution showed evidence of eosinophilic fasciitis, skin fibrosis with no evidence of myositis.

### Treatment plan

Patient was started on prednisone 20mg with taper over two months and stopped in parallel with methotrexate 15 mg once weekly for 2 weeks. Then methotrexate was increased to a maintenance dose of 20mg weekly afterwards with folic acid 5mg weekly. The patient presented a month later with marked improvement in skin findings. The itching improved and the myalgias decreased. His eosinophils dropped from 13.2% to 1%. His ESR, CRP, Cr, LFTs were normal, and creatinine was 1.1 mg/dL.

A month and a half after steroid taper, the patient started to have a recurrence of skin lesions. Prednisone was resumed with a taper from 20mg to a maintenance dose of 5mg daily with continuation of methotrexate. Three months later, there was diffuse skin thickening. Therefore, methotrexate was stopped and prednisone 20mg was resumed along with aporesidronate and vitamin D in addition to mycophenolic acid 500mg 2 tabs BID for 4 months. Unfortunately, despite these efforts, the treatment was not effective. Four months later, the patient reported no improvement but rather an aggravation of the symptoms over his abdomen and pelvis. Patient’s HbA1c became 8%. Then, the patient was given a rituximab infusion (1000mg) that was repeated in two weeks. Six months later, the patient reported no improvement and was unable to taper prednisone less than 20mg daily.

**Figure 2.**
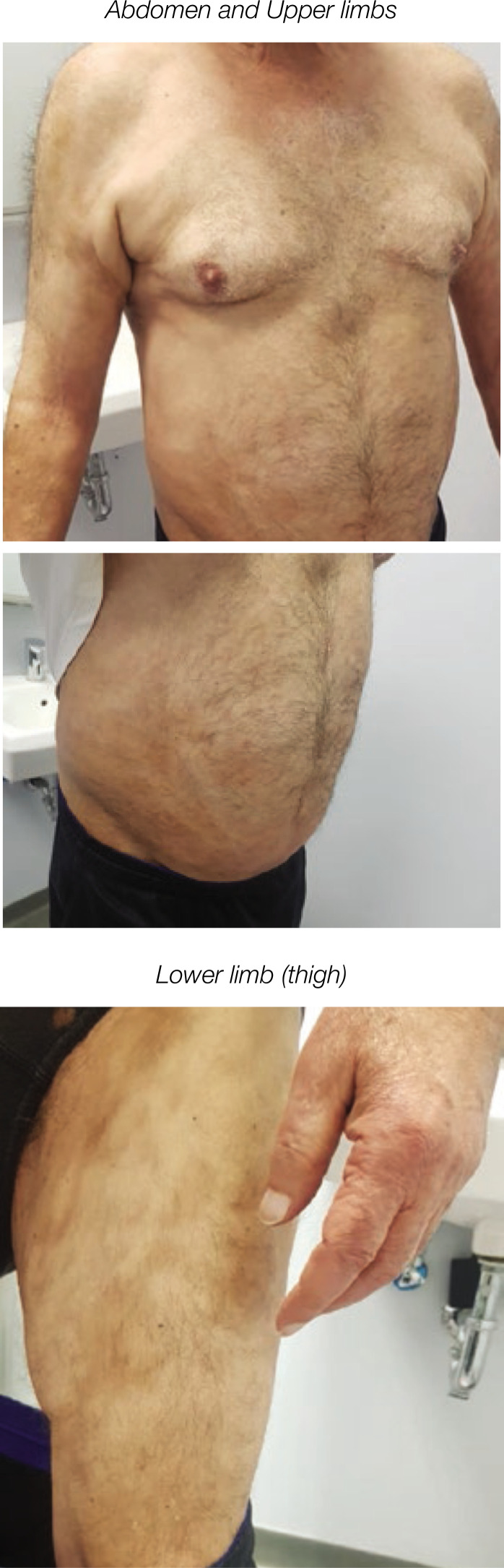
Physical exam findings after treatment.

Anti-IL5 therapy is not available in Lebanon. Thus, the patient was switched to Tocilizumab in February 2020, and within three months, he reported marked improvement while being off steroids. The patient no longer reported pruritus and his skin tightening has decreased (as shown in **Figure 2**). His labs, such as the complete blood count (including eosinophil count), lipid panel, CPK, PSA, TSH, vitamin D and B12 were all normal. On the physical exam, his skin appeared less tight and more elastic. Skin elasticity improved dramatically over his abdomen and thighs to date.

## REVIEW OF LITERATURE

### Pathophysiology

Although many experts view eosinophilic fasciitis as an autoimmune disease, the exact pathophysiology is still unclear. Several hypotheses have been postulated to explain the manifestations of the disease. The excessive fibrosis could be due to the increase in expression of dermal fibroblasts, type I collagen, and fibronectin in EF patients.^5^ The fibrosis could be generated by the elevated production of tissue inhibitor of metalloproteinase-1 (TIMP-1), an inhibitor of the extracellular matrix degrading enzyme matrix metalloproteinase-1 (MMP-1, collagenase).^6^ TIMP-1 was not only shown to be involved in the pathogenesis of the disease but might be a potential serological marker for disease activity.^6^ TIMP-1 level was directly correlated with a higher serum gamma globulin and IgG in EF patients. The increase in IgG level in EF patients was also a common finding in older studies.^2,7^ This led researchers to deal with EF as an autoimmune disease, especially with its coexistence with other auto-immune diseases such as autoimmune thyroiditis^8^ and response to corticosteroids.^9,10^

EF is associated with high levels of IL-5 which is a known stimulating factor and regulator of eosinophil build-up in tissues and is involved in its maturation and survival.^11^ It has been mentioned in literature that eosinophils play a key role in EF by degranulating and releasing cytokines such as IL-1, IL-6^11^, and TGF-B that stimulates fibroblasts.^11,12^ There is a great expression of TGFB-1 genes and Type 1 collagen in Fibroblasts seen in EF infiltrate.^11,12^

### Differential diagnosis: Difference between scleroderma and EF

Eosinophilic fasciitis (EF) is a rare inflammatory Fibrosing disease that mimics non-inflammatory Localized Scleroderma (morphea) in its fibrosing nature that can make it difficult to differentiate when presented clinically.^13,14^ Therefore, a definitive diagnosis can be determined by an en-bloc biopsy that can distinguish these two distinct connective tissue diseases.^1,3^ In EF, Fibrosis typically involves fascia and subcutaneous tissue beneath while in Morphea it is more likely to observe fibrosis in the dermis rather than in deeper fascia.^12^ Eosinophilic infiltrate can be seen in 50% of cases and the presence of peripheral eosinophilia makes it more likely to be EF.^1,12^

Patients presenting with EF are mostly ANA negative in contrast to scleroderma.^10^ Scleroderma is mainly a vasculopathy occurring in the capillaries which explains the visceral involvement while in EF, extracutaneous manifestation and Raynaud phenomenon are typically uncommon.^15^ However, there are reported cases of interstitial lung disease,^16^ pleuropericarditis,^17^ and renal disease.^18^ Eosinophilic fasciitis is usually bilateral and symmetrical while scleroderma tends to be unilateral with more defined borders.^12^ Distal extremities and finger sclerodactyly are present in scleroderma and absent in EF.^15^ Men are more affected with EF while scleroderma is more common among women.^12,19^

### Differential diagnosis: Considering other aetiologies

Other diagnosis (**Table 1**) may be excluded by the absence of certain features such as Raynaud phenomenon, abnormal capillaroscopy, and internal organ involvement.^4,14^ They can also be differentiated based on the characteristic distribution of cutaneous sclerosis, radiological, and tissue biopsy findings.^4,14^ Other diseases such as eosinophilia-myalgia syndrome are associated with systemic symptoms, including fever and myalgia, and typically present in epidemic form and seem to be toxin-associated (caused by L-tryptophane exposure).^14,20^

**Table 1.** Differential diagnosis of EF.

**Differential diagnosis^4,14,20^**
Scleroderma Systemic Sclerosis Localised Fibrosing Disorders - Linear Scleroderma, Morphea, and Regional Fibrosis Epidemic fasciitis syndromes caused by toxic agents such as the myalgia–eosinophilia syndrome Toxic Oil Syndrome Peripheral T cell lymphomas/paraneoplastic

## CRITERIA OF DIAGNOSIS AND DIAGNOSTIC TESTS

### Physical examination

Clinical findings that can be seen in Eosinophilic fasciitis are the “Peau d’orange” and the “Groove sign”.^21^ “Peau d ‘orange” or orange-peel skin is a characteristic finding caused by inflamed swelling and hardening of fibrosed muscular fascia.^4,21^ Additionally, the “Groove sign” is an area of deep depression that can be seen along veins that becomes more prominent when the affected limb is elevated.^4^

### Immunological studies

These findings are considered minor criteria for diagnosing EF and can be seen in more than half of patients: ESR, CRP, hypergammaglobulinemia, and eosinophilia^4^. Further, patients with EF are commonly ANA positive.^21^

### Imaging

MRI is a useful diagnostic tool to assess inflammation in the fascia, detect the best site to take biopsy, and for follow up. When MRI is not available, PET-Scan and ultrasound can be used.^22,23^

### Biopsy

Biopsy is always required for a definitive diagnosis as discussed in the diagnostic criteria below.^21^

### Diagnostic criteria

In Pinal-Frenaddez’s article “Diagnosis and Classification of Eosinophilic fasciitis” (2017), it was proposed that scleroderma should be first excluded. Then, EF is diagnosed when there are two major criteria, or one major and 2 minor criteria as listed below:

#### Major Criteria^4^

- Symmetric and asymmetric diffuse or localized swelling, induration and thickening of the skin and subcutaneous
- Histology showing fascial thickening with an accumulation of lymphocytes and macrophages with or without eosinophils

#### Minor Criteria^4^

- Peripheral eosinophilia (> 0.5 × 10^9^)
- Hypergammaglobulinemia (> 1.5 g/L)
- Muscular weakness and/or elevated serum aldolase
- Groove sign and/or Peau d ‘orange appearance of skin
- T2-weighted MRI showing hyperintense fascia

#### Exclusion Criteria^4^

##### Diagnosis of systemic scleroderma

It is important to mention that many physicians rely on wedge biopsy or MRI to fulfil the second major criterion.^21^ A year later, a diagnostic criterion along with a severity classification was proposed by the Japanese Dermatological Association in Jinnin et al.’s article in 2018.^24^ It was proposed that a definitive diagnosis of EF is made when a patient satisfies a major criterion and one of the minor criteria, or the major criterion and two of the minor criteria.

#### Major Criterion

Symmetrical plate-like sclerotic lesions are present on the four limbs. However, this condition lacks Raynaud’s phenomenon, and systemic sclerosis can be excluded.^24^

#### Minor Criteria 1

The histology of a skin biopsy that incorporates the fascia shows fibrosis of the subcutaneous connective tissue, with thickening of the fascia and cellular infiltration of eosinophils and monocytes.^24^

#### Minor Criteria 2

Thickening of the fascia is seen using imaging tests such as magnetic resonance imaging (MRI).^24^

#### Severity Classification of Eosinophilic Fasciitis^24^

-Joint contracture (upper limbs): 1 point
-Joint contracture (lower limbs): 1 point
-Limited movement (upper limbs): 1 point
-Limited movement (lower limbs): 1 point
-Expansion and worsening of Skin rash (progression of symptoms): 1 point

A total of 2 or more points is classified as severe.

### Common treatments

To date, the ideal approach for treating EF remains unclear due to disease rarity and lack of randomised controlled trials that tackle this disease. As per current practice, systemic corticosteroids remain the mainstay and first line treatment choice for EF by starting with doses equivalent to prednisone 1 mg/kg per day.^3,25^ In many patients, an improvement in skin findings occur and a quick decrease in eosinophil count and ESR is commonly witnessed.^12,25^ This is often followed by systemic glucocorticoid dose reduction.^3^ If symptoms and signs of EF as well as lab results fail to improve, the dose of systemic corticosteroids is usually gradually increased.^12,21,25^ However, when patients do not respond to initial therapy with systemic glucocorticoids or experience EF relapse, they require additional therapy.^12,21,25^ In particular, patients with an associated hematologic disease must be treated for the underlying disease.^12,21^

**Figure 3.**
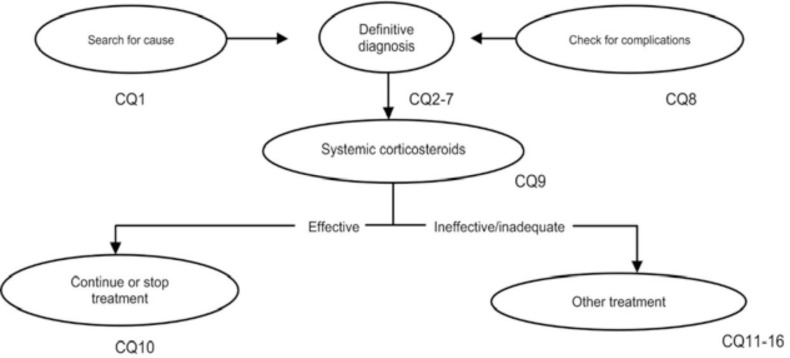
Algorithm by Jinnin et al., 2018.

The guidelines based on evidence-based medicine by the Japanese Dermatological Association published in “Diagnostic criteria, severity classification and guidelines of eosinophilic fasciitis” established this algorithm for treatment of eosinophilic fasciitis (**Figure 3**).^24^

The second line treatment for EF is the use of immunosuppressive agents such methotrexate, mycophenolate mofetil, cyclosporine, azathioprine, and cyclophosphamide.^21,26–32^ To date, the most used second line treatment used for EF is methotrexate with several studies suggesting its efficacy in adjunct to systemic corticosteroids even during initial treatment; however, the other immunosuppressive agents are still of limited evidence.^21,26,27^

There have been attempts to use monoclonal antibodies with various targets such as rituximab (Anti-CD20), tofacitinib, a JAK inhibitor targeting cytokine production pathway, reslizumab targeting IL-5, and tocilizumab targeting IL-6.^11,33–36^

## DISCUSSION OF SIMILAR PUBLISHED CASES

Recently, tocilizumaub is gaining interest among practitioners.^37^ Several case reports have shown the efficacy of Tocilizumab on non-RA connective tissues diseases, large-vessel vasculitis and others heterogeneous inflammatory conditions like Still’s disease, amyloidosis, scleroderma, Castleman’s disease, and most recently EF^38–41^ Tocilizumab is a humanized monoclonal antibody that binds to IL-6 receptor α and inhibits IL-6-mediated pro-inflammatory signaling.^27^ IL-6 is thought to stimulate collagen production and is involved in fibrosis pathophysiology in EF.^39^ Thus, an IL-6 inhibitor such as tocilizumab might play an essential role in halting progression and treat FE.^26,27^ It was first reported in 2015 that a patient who had EF refractory to corticosteroids, methotrexate and etanercept was found to respond to tocilizumab.^36^After this case report, several successful attempts for using Tocilizumab in corticosteroid resistant EF have been recorded in literature as shown in table below.^11,27,37^ Based on our search, the relevant case reports were published between 2015 and 2020 (**Table 2**). In all the cases, EF was refractory to systemic corticosteroids and methotrexate. Due to the persistence of EF symptoms, the authors decided to start the patients on tocilizumab. In 4 out of 5 cases in literature, the patient was maintained at the final stage on tocilizumab only like our patient, whereas in one case, a patient was maintained on combination therapy (tocilizumab + prednisone + methotrexate). Significant clinical response to tocilizumab was recorded in all cases.

**Table 2.** Case studies currently found in the literature that used tocilizumab for treating multiple regimen-resistant EF.

**Author**	**Patient**	**Initial Tx**	**Initial Response**	**Relapse**	**Second line TX**	**Response**	**Final Tx**
**Espinoza** (2015)	43 y.o. male	Prednisone	Yes	Yes	MTX and Etanercept	No	TCZ
**Vílchez-Oya** (2019)	60 y.o. male	Prednisone	No	N/A	MTX and IVIG	No	TCZ
**Urzal** (2019)	61 y.o. female	Prednisone	No	N/A	TCZ+ MTX + Prednisone	Yes	N/A
**Pinheiro** (2020)	37 y.o. male	Prednisone+MTX	Yes	Yes	MTX	Yes MTX stopped (Hepatotoxicity)	TCZ
**Pinheiro** (2020)	61 y.o. female	Prednisone + MTX	Yes	No, Steroids stopped (Avascular necrosis + Uncontrolled DM)	MTX	Partial response	TCZ
					**LEGEND**	TCZ: Tocilizumab MTX: Methotrexate Tx: Treatment DM: Diabetes Mellitus	

In our case report, our patient also failed corticosteroids, mycophenolate mofetil and methotrexate treatment. However, what makes our case different is that our patient is also resistant to rituximab, and he eventually improved on tocilizumab. This marks the sixth reported response to tocilizumab among EF patients recorded in literature. EF response to tocilizumab may be because eosinophilia and hypergammaglobulinemia in EF are explained by high production of IL-5 and IL-6 by mononuclear.^39^ In addition, eosinophils, which play a key role in EF, degranulate and release cytokines like IL-6 as explained before in “pathophysiology”.^5^

In summary, managing EF could be challenging due to its incomplete response or relapse. Although systemic corticosteroids remain the first line of treatment, several other treatments that are corticosteroid sparing options have shown to be effective. Even though data on tocilizumab is still limited to a few case reports, it might still be an effective and safe treatment for EF patients. Further investigation is needed to better understand the efficacy of tocilizumab in in patients who failed first- and second-line treatment, especially rituximab-resistant Eosinophilic fasciitis.
